# Care Integration – From "One Size Fits All" to Person Centred Care: Comment on "Achieving Integrated Care for Older People: Shuffling the Deckchairs or Making the System Watertight for the Future?"

**DOI:** 10.15171/ijhpm.2018.51

**Published:** 2018-06-18

**Authors:** Axel Kaehne

**Affiliations:** Evidence-Based Practice Research Centre, Edge Hill University, Ormskirk, UK.

**Keywords:** Integration, Partnership, Health Systems, Health Policy

## Abstract

Integrating services is a hot topic amongst health system policy-makers and healthcare managers. There is some evidence that integrated services deliver efficiencies and reduce service utilisation rates for some patient populations. In their article on *Achieving Integrated Care for Older People*, Gillian Harvey and her colleagues formulate some critical insights from practice and research around integrated care. However, the real challenge is to reconcile service integration with patient experiences. This paper argues that unless we think service integration from the patient’s perspective we will continue to fail to produce the evidence we need to support integrated care solutions to the current health system challenges.

In their article on *Achieving Integrated Care for Older People* Gillian Harvey and her colleagues formulate some critical insights from practice and research around integrated care.^1^ The specific focus of the article is the care for older people, yet the lessons they draw apply to other patient populations too. I would like to respond to the challenges that Harvey and colleagues identify by drawing out the main dilemma of integrated care, the tension between the organisational nature of integration programmes and their ultimate objective, the improvement of patient care.

The main issues when caring for older people relate to multi-morbidity, multi-pharmacy, and frequent hospitalisation. Seen through the lens of the triple aim, older people tend to receive poor quality of care, place an undue burden on health systems through high utilisation rates, and struggle to gain timely access to the right care. Within modern developed healthcare systems, several other patient populations are affected by similar difficulties.

It is widely agreed that integrating services potentially reduces some service utilisation and decreases costs in some circumstances.^2-4^ It is also thought to improve the experience of care as patients travel through the system, particularly for those with multiple healthcare needs.^5,6^ What appears plausible at a first glance however has been difficult to bring about. Truly integrated healthcare systems are rare and, where they occur, the cost savings are usually materialising only five to 8 years into the programme.^2,6,7^ In addition, there is some evidence that where integrated care provision operates it works best where patients have relatively few morbidities, incentivising providers to pick the low hanging fruit. Complex cases prove difficult to tackle even where professionals work in an integrated way. The sobering upshot is that integrated care has often remained an aspiration.

Harvey and colleagues rightly identify some of the important lessons from the current state of affairs. They question whether the one size fits all approach is justified when it comes to integrated care. They also advocate a shift in focus from organisations to patients and their care experience. Moreover, they call for more attention to implementing integrated care systems and identify what works for whom. And, finally, they also champion a bottom up rather than a top down approach. These lessons sound intuitively right and the conclusions of Harvey and her colleagues echo those of others. So why has there been so little progress in bringing about a more person centred way of delivering? I will try to formulate an answer to this question.

Medical care is at heart a rationalistic endeavour. Clinicians identify a problem and apply a solution that, ideally, is validated by scientific evidence. The way we structure our health systems is equally rationalistic. What strikes us as a system characterized by complexity is, at the policy level, one governed by rational decision making. We interrogate epidemiological data and examine patient flows to provide robust forecasts of patient demand. Each care organisation links with other organisations through care pathways, patient flows with associated tariffs linked by mutual governance arrangements. These organisations as a whole create a complex interrelated web of providers, the health system. On the other hand, the patient experience is constructed through a multitude of contingent impressions of care. At the heart of the care experience is a social interaction between patient and healthcare professionals. In addition, the patient experience occurs at both levels, at the single provider as well as at the system level.

Integration of care may take place within one organisation (clinical micro-systems) as well as in between organisations.^8^ This has created a fallacy of analogy. We somehow believe that integrating care is similar to improving care experiences. As Harvey et al. rightly point out, most integration programmes are implemented from the top down, with an institutional focus. The hope is that where integration occurs, patient care also improves. More recently, Singer and colleagues have argued that care integration also comprises normative and interpersonal dimensions.^9^ They note that the differentiation between the organisational and the social features of integration are essential to our understanding.

Current publications reflect this mistaken belief. Researchers design ever more sophisticated models to capture (and evaluate) the impact of integration. The latest fashion is the concept of complexity where collaborating professionals produce new organisational features (emergence) that are greater than the sum of their actions, activating feedback loops and creating non-linear causal chains.^10,11^ On the other hand, evaluation research increasingly accentuates interpretivist approaches, such as realist evaluation, reflecting misgivings about rigid positivist notions of knowledge. Ultimately, both complexity and realist research methodologies hope to achieve the same: to identify a model that tells us how the organisational map of integration aligns with the patient experience. But can they do this?

Harvey and colleagues make the important point that no single care experience is the same. And so no single person centred care plan could or should be the same. The care system thus needs to accommodate flexibility and contingency whilst being planned and designed along rationalistic models. I have discussed this elsewhere as an ambition of integrated care to be a scientific paradigm.^12^ I have argued that integrated care lacks one essential component to become a successful scientific paradigm: the patient experience. In its current form, integrated care does not conceptualise what constitutes good care as perceived by the patient in contradistinction from ordinary care. So far, integrated care is defined by what organisations do, not by how patients experience the care they receive. The hope of those who model integrated care is that there is an overlap between the two. Even if that was true, we would probably struggle to demonstrate when and why that is the case.

The way we construct knowledge in the field of integration tells us something about why there is no automatism in the desired confluence of organisational integration and patient experience. Services are designed in a rationalistic way whereas we talk about patient experience in an impressionistic way. In other words, the epistemological tools we bring to bear upon integrated care structures and the patient experience are mismatched. To be clear, we do know when patients experience good care. And we think we know what integrated care looks like but we have no instrumentarium to determine when, under what circumstances and why the two exist simultaneously, nor, if they did, whether or not they are associated. Does integrated care produce good patient experiences? And would patients, in the absence of a particular integrated type of provision, experience poorer care?

So why do we assume that integration and patient experience would coincide? The answer lies in the genesis of the discipline. More than two decades ago multiagency partnership work received considerable attention. The main concern was sectoral fragmentation in the care system.^13-16^ This was an issue of particular urgency for older people and people with chronic conditions who had to navigate various providers with different professional status, competencies and skills.

As time went by, the policy agenda around partnership gradually morphed into an integration issue. The motivation for integrating services was to create efficiencies in the system, ie, reduce the costs associated with demographic changes. In a sense, the partnership agenda was a health system policy for a time of plenty whereas integration reflected new needs in times of austerity.^17^ Care fragmentation came to be seen as a key barrier to deliver these efficiencies in the health system. The legacy of the work around partnership and overcoming fragmentation meant that an assumption built up that integrating services would necessarily lead to better patient experiences. Less fragmented services would surely be better for patients. Integration of services thus appeared to offer a metaphor for cost savings and better care quality, killing two birds with one stone. What remains weakly articulated in the new field of integrated care however is patient experience. This carries an epistemological dimension.

It seems to me that it is the discrepancy between patient experience and the organisational focus of most integrated care programmes that poses considerable policy and research challenges. In fact, I suspect that organisational or professional boundaries mean very little to patients. What matters is whether the journey of the patient is seamless and smooth through the system. If that is correct, then the way we currently approach integrated care is putting the cart before the horse, and no amount of patient consultation can change that. We have to start with the patient experience and work our way up rather than start with organisational dilemmas and hope for better patient care.

This will be challenging. Patients take multiple paths through the system. It is the essence of patient centred care that care experiences are tailored to individuals. Yet we can still plot typical journeys and ask how to reduce friction for patients along the way. We can then superimpose those experiential maps on to the organisational charts and interrogate the provider system. This way we can measure integration success through the lens of patients.

In 2001, a team led by Chad Boult investigated the effectiveness of an outpatient geriatric evaluation and management clinic on high risk older patients.^18^ Their primary outcome measure was functional ability of patients. The study continues to be cited in the field. The reason is simple. The study conceptualised the efficiency of services from the perspective of patients, their functional ability. At the heart of this is what patients want, championing a genuinely person centred approach. The authors interrogated which interventions would increase the effectiveness of services (defined by functional ability) and reduce costs by preventing disability among the population? It was a prime example of thinking about service efficiency with patients in mind. The paper shifts the focus from organisational priorities to patient experiences. It’s time we start to put the horse before the cart.

## Ethical issues

Not applicable.

## Competing interests

Author declares that he has no competing interests.

## Author’s contribution

AK is the single author of the paper.
